# Home-Based Palliative Care and Outcomes Among Persons Living With Dementia: A Systematic Review

**DOI:** 10.1093/geroni/igaf122.3179

**Published:** 2025-12-31

**Authors:** Xuefan Ji

**Affiliations:** Columbia University, New York, New York, United States

## Abstract

**Background:**

Persons living with dementia (PLWD) have high symptom burdens and significantly diminished quality of life due to the progressive nature of the disease. Palliative care is suitable for PLWD as it can provide symptom relief and comfort-based care throughout the disease trajectory. Home-based palliative care (HBPalC) is a model of care that expands palliative care access for PLWD as majority of PLWD live in their own homes.

**Aim:**

To systematically review and synthesize features of currently available HBPalC in use for PLWD and evaluate healthcare utilization and cost outcomes.

**Methods:**

PubMed, Embase and CINAHL were searched from inception until October 2024 and all U.S. based studies that included PLWD in patient samples and examined the effect of HBPalC on healthcare utilization and cost outcomes were included.

**Results:**

A total of seven studies were included, only two provided PLWD-specific HBPalC characteristics and outcomes. HBPalC interventions designed for PLWD involved addressing neurobehavioral symptoms. HBPalC was associated with fewer hospitalization, hospital length of stay, and inpatient and total costs for PLWD. Racial and ethnic PLWD were largely underrepresented in study samples.

**Discussion and Implications:**

More studies are needed to exclusively evaluate HBPalC’s impact on PLWD. There are gaps in knowledge about HBPalC’s impact on racial and ethnic minority PLWD healthcare utilization and cost outcomes. Future studies need to sample racial and ethnic minority PLWD intentionally and use more representative data to evaluate healthcare utilization and cost outcome data.

